# Cannabis, connectivity, and coming of age: Associations between cannabis use and anterior cingulate cortex connectivity during the transition to adulthood

**DOI:** 10.3389/fnhum.2022.951204

**Published:** 2022-11-11

**Authors:** Sarah D. Lichenstein, Daniel S. Shaw, Erika E. Forbes

**Affiliations:** ^1^Yale Imaging and Psychopharmacology (YIP) Lab, Department of Psychiatry, Yale School of Medicine, New Haven, CT, United States; ^2^Pitt Parents and Children Laboratory (PPCL), Department of Psychology, University of Pittsburgh, Pittsburgh, PA, United States; ^3^Affective Neuroscience and Developmental Psychopathology (ANDP) Lab, Department of Psychiatry, University of Pittsburgh, Pittsburgh, PA, United States

**Keywords:** marijuana, white matter, ACC, development, adolescence

## Abstract

Cannabis use is common among adolescents and emerging adults and is associated with significant adverse consequences for a subset of users. Rates of use peak between the ages of 18–25, yet the neurobiological consequences for neural systems that are actively developing during this time remain poorly understood. In particular, cannabis exposure may interfere with adaptive development of white matter pathways underlying connectivity of the anterior cingulate cortex, including the cingulum and anterior thalamic radiations (ATR). The current study examined the association between cannabis use during adolescence and emerging adulthood and white matter microstructure of the cingulum and ATR among 158 male subjects enrolled in the Pitt Mother and Child Project, a prospective, longitudinal study of risk and resilience among men of low socioeconomic status. Participants were recruited in infancy, completed follow-up assessments throughout childhood and adolescence, and underwent diffusion imaging at ages 20 and 22. At age 20, moderate cannabis use across adolescence (age 12–19) was associated with higher fractional anisotropy (FA) of the cingulum and ATR, relative to both minimal and heavy adolescent use. Longitudinally, moderate and heavy extended cannabis use (age 12–21) was associated with reduced positive change in FA in the cingulum from age 20 to 22, relative to minimal use. These longitudinal results suggest that cannabis exposure may delay cingulum maturation during the transition to adulthood and potentially impact individuals’ functioning later in development.

## Introduction

Cannabis is currently the most widely used illicit drug of abuse, with 49% of adults in the United States reporting lifetime use (SAMHSA, 2022). Rates of use are particularly high among adolescents and young adults, with approximately 23% of individuals in the US aged 18–25 reported cannabis use in the last month (SAMHSA, 2022). Despite growing public perception of cannabis as benign (Johnston et al., 2022), cannabis use can have significant deleterious consequences for some users, including substance dependence, mental health problems, alterations in neural structure and function, and poor psychosocial attainment (Volkow et al., 2014; Hill et al., 2022; Lichenstein et al., 2022). Identifying individuals who are at the highest risk for these negative consequences could facilitate the development of targeted prevention and intervention programs to mitigate cannabis’ long-term deleterious impacts. Therefore, it is imperative to elucidate the mechanisms underlying the adverse consequences of cannabis use to predict how and for whom cannabis use is most likely to lead to negative long-term outcomes.

White matter is thought to be an important target of cannabis effects on the brain during adolescence and emerging adulthood (Lichenstein et al., 2022). Comprised of myelinated axon bundles (known as white matter pathways or tracts), white matter provides the structural basis for neural signaling and undergoes significant developmental changes into early adulthood (Paus, 2010; Westlye et al., 2010; Simmonds et al., 2014). Chronic cannabis exposure is associated with a downregulation of endogenous cannabinoid (CB1) receptors in the brain (Hirvonen et al., 2012). Such downregulation may interfere with normative endocannabinoid functioning and negatively impact white matter integrity *via* increased neuroinflammation (Verstynen et al., 2013; Tian et al., 2014; Bettcher et al., 2015; Morena et al., 2015), reduced oligodendrocyte survival (Molina-Holgado et al., 2002; Bortolato et al., 2014) and/or decreased myelination (Lubman et al., 2015).

In particular, white matter pathways that support connectivity of the anterior cingulate cortex (ACC) may be critical targets of cannabis effects during the transition to adulthood. The ACC plays an important role in integrating cognitive, affective, and social neural networks to guide behavior (Fossella et al., 2008), and has been hypothesized to function as a hub for internetwork connectivity (Luna et al., 2015; Lichenstein et al., 2016). Although the basic architecture of ACC connectivity remains stable from childhood, changes in the strength of various white matter pathways facilitate specialization and integration of neural networks across adolescence (Luna et al., 2015). Specifically, the cingulum and anterior thalamic radiations (ATR) are the primary white matter pathways linking the ACC with its distributed cortical and subcortical targets. Each of these pathways follows a protracted developmental course, reaching estimated peaks at ages 34 and 28, respectively (Lichenstein et al., 2016). Thus, the developmental period of greatest prevalence of problem-level cannabis use coincides with ongoing development in the structural connections that facilitate sophisticated, circuit-based functioning of the brain.

Anterior cingulate cortex connectivity may be particularly sensitive to the effects of cannabis exposure during the transition into adulthood. Chronic cannabis users exhibit a particularly marked downregulation of CB1 receptor density in the ACC and neocortex (Hirvonen et al., 2012), and late adolescent/young adult cannabis users exhibit altered local network organization of the cingulate cortex relative to controls (Kim et al., 2011). Converging evidence across cross-sectional (Delisi et al., 2006; Bava et al., 2009; Gruber et al., 2011, 2014; Jacobus et al., 2013c; Shollenbarger et al., 2015; Jakabek et al., 2016; Wade et al., 2020; Courtney et al., 2022) and longitudinal (Bava et al., 2013; Jacobus et al., 2013a,b; Becker et al., 2015) studies indicate altered cingulum and ATR microstructure among cannabis users. However, cross-sectional studies report both increased and decreased white matter integrity, and existing longitudinal reports are limited based on small sample sizes (maximum *N* = 48 to date) and inconsistency in the age range studied. The current study builds upon previous literature by utilizing a large sample of low income, urban men, a population with particularly high rates of cannabis use. Additionally, we targeted the transition to adulthood by analyzing changes in the cingulum and ATR from ages 20 to 22, allowing us to examine longitudinal associations between cannabis use and developing white matter.

Specifically, the objectives of the current study were to examine the association between cannabis use and: (1) ACC connectivity (cingulum and ATR microstructure) at age 20 and (2) developing ACC connectivity (change in cingulum and ATR microstructure) from ages 20 to 22. We hypothesized that: (1) adolescent cannabis use (age 12–19) would predict poorer white matter integrity at age 20 [i.e., lower fractional anisotropy (FA)], and (2) extended cannabis use across adolescence and the transition to adulthood (age 12–21) would be associated with less positive change in FA from ages 20 to 22 in the cingulum and ATR.

## Materials and methods

The current sample (*n* = 158) was 51.3% European American, 41.1% African American and 7.6% other races (see Table 1 for subject characteristics). Participants were characterized by low family income in early childhood and less than 14% reported a non-substance-related psychiatric disorder at age 20 or 22.

**TABLE 1:** Sample demographic and clinical characteristics.

			Extended cannabis use group							
			
	Full sample (*n* = 158)		Minimal/no cannabis exposure (*n* = 53)		Moderate cannabis exposure (*n* = 52)		Heavy cannabis exposure (*n* = 53)		Group comparison	
					
	*N*	%	*N*	%	*N*	%	*N*	%	χ^2^	*p*
**Race**										
White	81	51.3	32	60.4	24	46.2	25	47.2	5.87	0.437
Black	65	41.1	16	30.2	24	46.2	25	47.2		
Biracial	8	5.1	3	5.7	2	3.8	3	5.7		
Other	4	2.53	2	3.8	2	3.8	0	0		

	***M***	**SD**	***M***	**SD**	***M***	**SD**	***M***	**SD**	***F***	***p***

**SES**										
Family income (per month) (mean first 3 assessments)	1,208.18	669.9	1,236.88	594.24	1,409.28^(3)^	768.12	982.16^(2)^	574.26	5.74	0.004**
Neighborhood risk score (mean first 3 assessments)	0.37	1.12	0.03^(3)^	0.81	0.45	1.25	0.63^(1)^	1.2	4.06	0.019*
**Childhood clinical and cognitive assessments**										
Internalizing Symptoms (Mean Age 10-12)	5.28	4.99	5.38	5.14	4.95	4.82	5.49	5.07	0.55	0.581
Externalizing symptoms (mean age 10–12)	8.65	6.85	7.98	6.51	7.77	6.22	10.09	7.55	0.15	0.865
IQ (age 11)	96.11	18.39	97.89	20.82	96.61	19.94	93.86	13.94	1.68	0.19

*p < 0.05; **p < 0.01. Superscript numbers in parentheses indicate which groups were significantly different from one another, based on pairwise Bonferroni-corrected *post hoc* testing or pairwise χ^2^ tests, as applicable (1 = minimal/no cannabis exposure group, 2 = moderate cannabis exposure group, 3 = heavy cannabis exposure group). Race was assessed by self-report: participants indicated whether they identified as Asian, Black/African American, Caucasian/White, Hispanic, Mexican American, Native American, Native Hawaiian, Biracial, or other. Subjects included in the current analyses endorsed four different racial classifications, Black, White, Biracial, or other, and data were not further reduced. Internalizing and externalizing symptoms were measured using the Child Behavior Checklist (CBCL) (Achenbach and Edelbrock, 1983), parent report, at child age 10, 11, and 12. IQ was assessed using a short form of the Wechsler Intelligence Scale for Children (WISC-III) (Wechsler, 1991).

### Pitt Mother and Child Project

Participants were part of the Pitt Mother and Child Project (PMCP; Shaw et al., 2003), a longitudinal study of risk and resilience among men from families of low socioeconomic status (SES). A total of 310 mother-son dyads were recruited from Women, Infant, and Children (WIC) Nutritional Supplement centers in the Pittsburgh metropolitan area when subjects were 6–17 months old. After being initially assessed at either 12 or 18 months of age, they were followed throughout childhood, adolescence, and into young adulthood (in-person home, lab, and/or internet/phone assessments at ages 1.5, 2, 3.5, 5, 5.5, 6, 8, 10, 11, 12, 15, 16, 17, 18, 20, 21, 22, and 23) (Shaw et al., 2003). All procedures were approved by the Institutional Review Board at the University of Pittsburgh, with all assessments performed in accordance with relevant guidelines and regulations.

Participants were excluded from the magnetic resonance imaging (MRI) portion of the study if they endorsed any standard MRI contraindications (e.g., presence of metal in body). Out of the full sample (*N* = 310), *n* = 186 completed an MRI scan at age 20 (*n* = 31 declined, *n* = 17 could not be contacted, *n* = 10 were incarcerated, *n* = 5 lived out of the area, *n* = 5 were in the military, *n* = 1 was deceased, and *n* = 55 endorsed contraindications to MRI). Of those completing the scan at age 20, 28 did not complete a second scan, resulting in a subset of *n* = 158 male PMCP participants for whom good-quality diffusion tensor imaging (DTI) data were acquired at both ages 20 and 22.

### Measures

#### Cannabis use

Lifetime cannabis use was assessed with the Lifetime History of Drug Use and Drug Consumption (LHDU; Skinner, 1982; Day et al., 2008) semi-structured interview at ages 20 and 22. Participants who endorsed a positive lifetime history of cannabis use (at least 3 times in 1 year) reported their age of cannabis use onset, annual frequency of use, and their greatest use in one day for each year since their first use.

Adolescent cannabis use was quantified by calculating the sum of participants’ average days of use/month at each time point from ages 12 to 19. As participants were scanned around their 20th birthday, age 19 represents the year preceding their baseline DTI scan. As the data were not normally distributed and contained a significant proportion of zero values, the assumptions of a conventional linear regression were violated. Based on the distribution of the data and published recommendations (Boulton and Williford, 2018), we opted to transform the continuous data into discrete categories. Therefore, the sample was divided into terciles based on total frequency of use from ages 12 to 19, which consisted of a minimal adolescent use group (*n* = 56; ≤1 days/month), a moderate adolescent use group (*n* = 49; ∼weekly use), and a heavy adolescent use group (*n* = 53; multiple days of use/week).

Extended cannabis use across adolescence and the transition to adulthood was measured by calculating the sum of participants’ average days of use/month at each time point from ages 12 to 21. As participants were scanned around their 22nd birthday, age 21 represents the year preceding their follow-up DTI scan. Again, participants were split into terciles, with *n* = 53 participants assigned to a minimal extended use group (<1 day/week), *n* = 52 assigned to a moderate extended use group (<1.5 days of use/week), and *n* = 53 assigned to a heavy extended use group (multiple days of use/week). The majority of participants displayed a consistent level of use between adolescence and the transition to adulthood, such that *n* = 139 participants (88% of the sample) were classified into the same group based on their pattern of use across adolescence and their extended pattern of use across adolescence and the transition to adulthood. Very few (*n* = 19, 12%) shifted use groups: eight participants decreased their use between adolescence and the transition to adulthood, transitioning from the heavy adolescent use group into the moderate extended use group. Eleven participants increased their use, with 3 transitioning from the low adolescent use group to the moderate extended use group and 8 transitioning from the moderate adolescent use group to the heavy extended use group.

As data were collected on cannabis use across adolescence and extending into the transition to adulthood, we chose to focus our primary analyses on participants’ overall quantity of cannabis use (above). This strategy allowed us to incorporate the most data and to account for individuals’ overall level of exposure across development. Nonetheless, the richness of the PMCP dataset also allowed us to examine associations with age of onset, chronicity of use, and recent frequency of use. Age of onset of cannabis use was assessed with the LHDU (Skinner, 1982; Day et al., 2008) semi-structured interview. Chronicity of use was calculated as the number of years during which participants endorsed using cannabis at least 1 day/month from age 12 through age 19. Recent frequency of use was defined as participants’ average days/month using cannabis during the past year (age 19 for cross-sectional analyses; age 21 for longitudinal analyses).

#### Alcohol use

Alcohol use was assessed with the Lifetime History of Alcohol Use and Alcohol Consumption semi-structured interview (Skinner, 1982) at age 20. Alcohol use frequency and number of drinks were multiplied to obtain a measure of overall quantity of alcohol exposure for each year since first use. Alcohol exposure for each year from ages 13 to 19 was summed to create a measure of lifetime alcohol exposure for cross-sectional analyses. Alcohol exposure for each year from ages 13 to 21 was summed to create a measure of lifetime alcohol exposure for longitudinal analyses. Both variables were log transformed to account for a positive skew in the data.

#### Tobacco use

Each participant was classified based on whether or not they reported daily use of tobacco during the last year on The Alcohol and Drug Consumption Questionnaire (ADCQ; Cahalan, Cisin, and Crossley) at ages 20 and age 22.

#### Other illicit substance use

Lifetime history of illicit substance use was assessed using the LHDU (Skinner, 1982; Day et al., 2008) semi-structured interview at ages 20 and 22, including cocaine/crack, stimulants, sedative, opioids, inhalants, hallucinogens, and ecstasy. Positive lifetime history was determined based by consensus from age 20 and age 22 study visits.

#### Race

Self-report of race at age 20 was used. Participants reported whether they identified as Asian, Black/African American, Caucasian/White, Hispanic, Mexican American, Native American, Native Hawaiian, Biracial, or other.

#### Socioeconomic status

The current study used a composite measure including both familial income and neighborhood adversity, averaged across the first three assessments (ages 1.5, 2, and 3.5). Family income was assessed by mother report. Neighborhood adversity was quantified by combining several block level variables from census data collected in 1990 (Shaw et al., 2012). Both family income and neighborhood adversity were converted to *Z*-scores, and the mean of these standardized scores was used as the composite measure of early childhood SES.

#### IQ

Prorated Full Scale IQ (FSIQ) scores were derived from participants’ performance on a short form of the Wechsler Intelligence Scale for Children (WISC-III; Wechsler, 1991) at age 11.

#### Early adolescent problem behavior

Internalizing and externalizing scores from the parent report form of the Child Behavior Checklist (Achenbach and Edelbrock, 1983) were averaged across assessments at child ages 10, 11, and 12, as these assessments precede the onset of cannabis use. Both variables were log transformed to account for positive skew in the data, which is typical for internalizing and externalizing scores on the CBCL at this age period.

#### Head motion

Mean head displacement was calculated for each participant for each DTI scan.

### Diffusion tensor imaging

Participants underwent DTI scanning at age 20 and 22 on a 3T Siemens Tim Trio scanner at the University of Pittsburgh MR Research Center.

#### Diffusion tensor imaging acquisition

Two axial 2D DTI bipolar scans were acquired using identical parameters at both ages: time-to-repetition (TR) = 8,400 ms; time-to-echo (TE) = 91 ms; field of view = 256 × 256; frequency = 96; phase = 96; 64 slices of 2 mm thickness were acquired for a total scan time = 9 min and 56 s. Diffusion-sensitizing gradient encoding was applied in 61 uniform angular directions with a diffusion weighting of *b* = 1,000 s/mm^2^. Seven reference images with no diffusion gradient (*b* = 0) were also acquired.

#### Diffusion tensor imaging preprocessing

Preprocessing was carried out using the Oxford Centre for Functional MRI of the Brain (FMRIB) Software Library (FSL) (Smith et al., 2004) using tract-based spatial statistics (TBSS) (Smith et al., 2006), including brain extraction, eddy current correction, and fitting a tensor model at each voxel. All subjects’ FA data were eroded, end slices were removed to eliminate likely outliers, and non-linear registration was used to align all FA images into a common space (Rueckert et al., 1999; Andersson et al., 2010). A mean FA image was then created and thinned to generate a mean FA skeleton onto which each subject’s aligned FA data were then projected. For mean (MD), axial (AD), and radial (RD) diffusivity, the mean image was registered to the FA skeleton (i.e., as our hypotheses focus on FA). FA results are presented in the main body of the text and MD, AD, and RD results are presented in Supplementary Table 2). The Johns Hopkins University White Matter Tractography Atlas (Wakana et al., 2007) was used to identify the right and left cingulum (cingulate gyrus) and ATR as regions of interests (ROIs). Finally, fslmaths was used extract each ROI and fslmeants was used to calculate mean FA for each subject within each ROI of the skeletonized data in standard space. Mean FA, MD, AD, and RD values for each ROI were extracted to SPSS for further analysis. The cingulate gyrus ROI includes fibers that travel through coronal planes at both the middle of the splenium of the corpus callosum and the middle of the genu of the corpus callosum. The ATR ROI includes fibers that travel through coronal planes at the middle of the genu of the corpus callosum and the thalamus, excluding any fibers that cross the corpus callosum (Wakana et al., 2007).

### Data analysis

Analysis of covariance (ANCOVA) was used to determine whether microstructure of the cingulum and ATR at age 20 differed between adolescent cannabis use groups. Separate models were constructed for FA, MD, AD, and RD of the cingulum and ATR. Alcohol use, tobacco use, IQ, SES, and child problem behavior were considered as covariates based on reported associations with measures of white matter integrity. To determine the appropriate covariates to include in the primary models, the Akaike information criterion (AIC) was used to compare candidate models and any covariate that substantially improved the model fit (difference in AIC ≥2) was included in the final analyses (see Supplementary Table 1). Based on these analyses, no proposed covariates significantly improved the model fit so they were not included in the primary models. Hemisphere and head motion were included as covariates in all analyses (Yendiki et al., 2014), and subject ID number was included as a random effect variable. To account for computing these analyses for both ROIs, main effects were considered significant at a Bonferroni corrected threshold of *p* < 0.025 and Bonferroni-corrected *post hoc* pairwise tests were used to probe significant main effects.

An exploratory whole-brain analysis was also performed using the *randomise* tool in FSL (Winkler et al., 2014) to assess associations between adolescent cannabis exposure and white matter microstructure throughout the brain. A voxel-based FWE-corrected significance threshold of *p* < 0.01 was used to evaluate results. Any additional regions identified in which age 20 microstructure differed significantly between adolescent use groups were also to be included in subsequent longitudinal analyses.

Analysis of covariances were also computed to estimate whether change in microstructure of the cingulum or ATR from ages 20 to 22 (i.e., difference score for FA from age 20 to 22) varied between extended cannabis use groups. Again, to account for computing these analyses for both ROIs, main effects were considered significant at a Bonferroni corrected threshold of *p* < 0.025. To ensure that these results were not biased by the subsample of participants whose pattern of use changed substantially between adolescence and the transition to adulthood (*n* = 19 participants were classified into a different cannabis use group based on their use during adolescence versus their use across adolescence and extending into emerging adulthood, see section “Cannabis use” above), these analyses were repeated including only those participants who displayed a consistent pattern of use across adolescence and the transition to adulthood (*n* = 139).

Finally, linear regression analyses were conducted to examine associations between other cannabis use characteristics – age of onset, chronicity of use, and recent frequency of use – and age 20 FA and change in FA from ages 20 to 22, controlling for head motion.

#### Sensitivity analyses

Recent data suggests that cannabis effects on cingulum microstructure may only be evident when cannabis is used with nicotine (Courtney et al., 2022). Additionally, there is also evidence that accounting for alcohol use may attenuate cannabis associations with brain structure (Weiland et al., 2015), and that cannabis and alcohol may have interactive effects on FA of the cingulum and ATR (Wade et al., 2020). Therefore, we conducted additional sensitivity analyses to determine whether the pattern of results observed in our primary analyses was driven by tobacco or alcohol use. Accordingly, our primary analyses examining the association between adolescent cannabis use group and age 20 FA and the association between extended cannabis use group and change in FA from ages 20 to 22 were repeated controlling for tobacco and alcohol use. Additionally, we also repeated our primary analyses after excluding daily tobacco users.

## Results

### Subject characteristics

#### Cannabis use

Consistent with the high prevalence of use among men of low SES (Carliner et al., 2017), 79% of participants (*n* = 124) reported a lifetime history of cannabis use [compared with 52.7% in a nationally representative sample (SAMHSA, 2016)]. No participants reported regular use prior to age 12 (see Table 2 for details on cannabis use).

**TABLE 2:** Cannabis use characteristics.

			Extended cannabis use group							
			
	Full sample (*n* = 158)		Minimal/no cannabis exposure (*n* = 53)		Moderate cannabis exposure (*n* = 52)		Heavy cannabis exposure (*n* = 53)		Group comparison	
					
	*M*	SD	*M*	SD	*M*	SD	*M*	SD	*F*	*p*
Age of onset (*n* = 124 lifetime users)	15.74	2.16	17.73^(2,3)^	1.4	16.12^(1,3)^	2.12	14.68^(1,2)^	1.81	19.14	<0.001**
Duration of use (*n* = 124 lifetime users)	4.44	2.75	0.26^(2,3)^	0.56	3.77^(1,3)^	1.96	6.58^(1,2)^	1.57	109.83	<0.001**
**Average frequency of cannabis use (days/month; *n* = 124 lifetime users)**										
Age 12	0.42	3.09	0	0	0.02	0.14	0.96	4.69	1.45	0.239
Age 13	0.84	3.77	0	0	0.12	0.51	1.87	5.65	3.47	0.034*
Age 14	2.02	5.8	0^(3)^	0	0.55^(3)^	2.8	4.27^(1,3)^	8.02	7.36	0.001**
Age 15	3.96	8.42	0^(3)^	0	1^(3)^	3.17	8.29^(1,3)^	11.14	15.192	<0.001**
Age 16	6.73	10.46	0^(3)^	0.02	3.74^(3)^	7.47	12.29^(1,3)^	12.25	16.68	<0.001**
Age 17	8.06	11.28	0.07^(3)^	0.23	3.13^(3)^	6.5	16.07^(1,3)^	12.23	35.52	<0.001**
Age 18	10.64	12.29	0.06^(3)^	0.1	4.52^(3)^	7.14	20.63^(1,3)^	11.35	60.63	<0.001**
Age 19	11.28	12.43	0.09^(3)^	0.24	4.02^(3)^	5.18	22.42^(1,3)^	10.68	96.95	<0.001**
Age 20	11.68	12.69	0.14^(3)^	0.33	3.84^(3)^	5.29	22.97^(1,3)^	10.39	101.95	<0.001**
Age 21	9.57	11.67	0.07^(3)^	0.23	3.39^(3)^	4.52	18.93^(1,3)^	11.84	59.95	<0.001**

	***N***	**%**	***N***	**%**	***N***	**%**	***N***	**%**	**χ ^2^**	***p***

**Substance use disorders at age 20**										
Substance abuse	33	20.9	0^(2,3)^	0	7^(1,3)^	4.43	26^(1,2)^	16.46	40.38	<0.001**
Cannabis abuse	31	19.6	0^(2,3)^	0	5^(1,3)^	3.16	26^(1,2)^	16.46	45.36	<0.001**
Substance dependence	21	13.3	0^(2,3)^	0	4^(1,3)^	2.53	26^(1,2)^	10.76	25.47	<0.001**
Cannabis dependence	18	11.4	0^(3)^	0	2^(3)^	1.27	26^(1,2)^	10.13	28.3	<0.001**
**Substance use disorders at age 22**										
Substance abuse	39	24.7	0^(2,3)^	0	10^(1,3)^	19.2	29^(1,2)^	54.7	44.51	<0.001**
Cannabis abuse	39	24.7	0^(2,3)^	0	11^(1,3)^	21.2	28^(1,2)^	52.8	40.62	<0.001**
Substance dependence	14	8.9	0^(3)^	0	1^(3)^	1.9	13^(1,2)^	24.5	24.64	<0.001**
Cannabis dependence	13	8.2	0^(3)^	0	0^(3)^	0	13^(1,2)^	24.5	28.06	<0.001**

*p < 0.05; **p < 0.01. Superscript numbers in parentheses indicate which groups were significantly different from one another, based on pairwise Bonferroni-corrected *post hoc* testing or pairwise χ^2^ tests, as applicable (1 = minimal/no cannabis exposure group, 2 = moderate cannabis exposure group, 3 = heavy cannabis exposure group). Duration of use reflects the number of years (from age 12 to 21) that participants reported cannabis use frequency ≥1×/week. Substance use disorder diagnoses determined based on the Structured Clinical Interview for DSM-IV (SCID), administered at age 20 and 22 study visits. Substance abuse: includes cannabis abuse (age 20 *n* = 31, age 22 *n* = 39), sedative abuse (age 20 *n* = 0, age 22 *n* = 4), stimulant abuse (age 20 *n* = 0, age 22 *n* = 1), opioid abuse (age 20 *n* = 2, age 22 *n* = 3), cocaine abuse (age 20 *n* = 2, age 22 *n* = 2), hallucinogen/PCP abuse (age 20 *n* = 2, age 22 *n* = 2), and other substance abuse (age 20 *n* = 1, age 22 *n* = 1). Substance dependence: includes cannabis dependence (age 20 *n* = 18, age 22 *n* = 13), sedative dependence (age 20 *n* = 1, age 22 *n* = 2), opioid dependence (age 20 *n* = 1, age 22 *n* = 3), cocaine dependence (age 20 *n* = 1, age 22 *n* = 1), hallucinogen/PCP dependence (age 20 *n* = 0, age 22 *n* = 1), and other substance dependence (age 20 *n* = 1, age 22 *n* = 0).

#### Alcohol and other illicit substance use

Ninety-six percent of participants (*n* = 151) reported a lifetime history of alcohol use. Cumulative alcohol exposure was higher among those with higher rates of cannabis use. Less than 15% of participants reported lifetime use of an illicit drug other than cannabis (see Table 3 for additional information on alcohol and other substance use).

**TABLE 3:** Alcohol and other substance use characteristics.

			Extended cannabis use group							
			
	Full sample (*n* = 158)		Minimal/no cannabis exposure (*n* = 53)		Moderate cannabis exposure (*n* = 52)		Heavy cannabis exposure (*n* = 53)		Group comparison	
					
	*M*	SD	*M*	SD	*M*	SD	*M*	SD	*F*	*p*
Cumulative alcohol exposure	86.01	176.18	24.76^(3)^	65.76	68.57^(3)^	100.76	1485.23^(1,2)^	262.65	9.5	<0.001**

	***N***	**%**	***N***	**%**	***N***	**%**	***N***	**%**	**χ ^2^**	***p***

Daily tobacco smoker (age 20)	44	27.8	3^(2,3)^	5.7	14^(1,3)^	26.9	27^(1,2)^	50.9	25.76	<0.001**
Daily tobacco smoker (age 22)	46	29.1	3^(2,3)^	5.7	13^(1,3)^	25	30^(1,2)^	56.6	33.96	<0.001**
**Lifetime history of illicit substance use**										
Cocaine/crack	16	10.1	0^(2,3)^	0	4^(1,3)^	7.7	12^(1,2)^	22.6	15.43	<0.001**
Stimulants	13	8.2	1	1.9	5	9.6	7	13.2	4.7	0.096
Sedatives	20	12.7	1^(3)^	1.9	5^(3)^	9.6	14^(1,2)^	26.4	15.07	0.001**
Opioids	21	13.3	1^(3)^	1.9	5^(3)^	9.6	15^(1,2)^	28.3	16.95	<0.001**
Inhalants	5	3.2	0	0	1	1.9	4	7.5	5.32	0.07
Hallucinogens	19	12	0^(2,3)^	0	7^(1)^	13.5	12^(1)^	22.6	12.99	0.002**
Ecstasy	22	13.9	0^(2,3)^	0	5^(1,3)^	9.6	17^(1,2)^	32.1	23.95	<0.001**

*p < 0.05; **p < 0.01. Superscript numbers in parentheses indicate which groups were significantly different from one another, based on pairwise Bonferroni-corrected *post hoc* testing or pairwise χ^2^ tests, as applicable (1 = minimal/no cannabis exposure group, 2 = moderate cannabis exposure group, 3 = heavy cannabis exposure group). Cumulative alcohol exposure reflects the sum of participants’ annual quantity of alcohol use (average days/month and average drinks/occasion were multiplied in order to obtain a measure of overall quantity of alcohol exposure for each year). Lifetime history of illicit substance use was assessed using the Lifetime History of Drug Use and Drug Consumption (LHDU) semi-structured interview; positive lifetime history was determined based by consensus from age 20 and age 22 study visits.

### Cross-sectional association between adolescent cannabis use and anterior cingulate cortex connectivity at age 20

#### Cingulum

**FIGURE 1:**
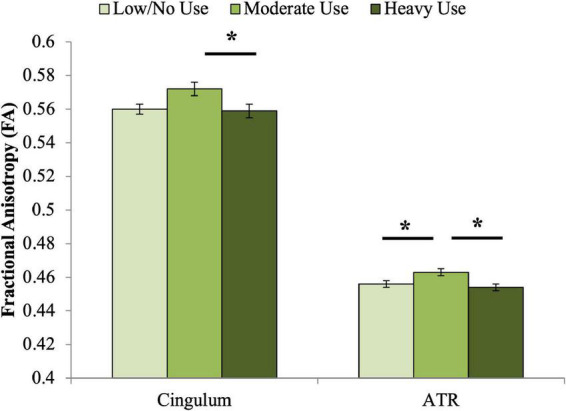
Associations between adolescent cannabis exposure and FA of the cingulum and ATR at age 20. Significant associations were observed between adolescent cannabis exposure group and FA of both the cingulum and anterior thalamic radiations. For the cingulum, Bonferroni-corrected pairwise *post hoc* tests demonstrated that FA of the moderate adolescent use group was significantly higher than the heavy adolescent use group (*p*_corrected_ < 0.05). For the anterior thalamic radiations, Bonferroni-corrected pairwise *post hoc* tests demonstrated that the moderate use and low or no use groups differed significantly (*p*_corrected_ < 0.05), as did the moderate and heavy use groups (*p*_corrected_ < 0.01). *indicates significant Bonferroni-corrected *post hoc* pairwise tests.

A significant association was observed between adolescent cannabis use group and cingulum FA at age 20 (Table 4 and Figure 1). Contrary to our hypothesis, the moderate use group displayed higher FA than both other groups. Bonferroni-corrected pairwise *post hoc* analyses demonstrated that FA of the moderate adolescent use group was significantly higher than the heavy adolescent use group (*p*_corrected_ = 0.013).

**TABLE 4:** Associations between cannabis use and FA of the cingulum and ATR.

	Cingulum			ATR		
		
	*F*	*df*	*p*	*F*	*df*	*p*
**Age 20 FA^•^**						
Adolescent cannabis use group	**3.83**	**2**	**0.023**	**6.41**	**2**	**0.002**
Hemisphere	**11.83**	**1**	**< 0.001**	**15.88**	**1**	**< 0.001**
Head motion	5.07	1	0.025	0.49	1	0.483
**Change in FA from 20 to 22^••^**						
Extended cannabis use group	**4.43**	**2**	**0.013**	*3.48*	*2*	*0*.*032*
Hemisphere	**15.52**	**1**	**< 0.001**	2.64	1	0.105
Head motion (age 20)	3.08	1	0.08	1.53	1	0.217
Head motion (age 22)	1.96	1	0.162	1.38	1	0.242

Each quadrant represents one ANCOVA and significant associations (*p* < 0.025) are bolded. Trend level findings are italicized. ^•^df_error_ = 311, df_total_ = 316; ^••^df_error_ = 310, df_total_ = 316. Change in FA from 20 to 22 represents the difference score for FA from age 20 to 22. FA, fractional anisotropy; ATR, anterior thalamic radiations; *df*, degrees of freedom.

#### Anterior thalamic radiations

A significant association was also observed between adolescent cannabis use group and ATR FA (Table 4 and Figure 1). Similar to the pattern observed for cingulum FA, the moderate adolescent use group displayed significantly higher FA than both the minimal and the heavy adolescent use groups (*p*_corrected_’s < 0.05).

#### Whole-brain results

The whole-brain analysis did not reveal any clusters throughout the white matter skeleton in which white matter microstructure differed significantly between adolescent cannabis use groups. Therefore, subsequent analyses of associations between cannabis use and longitudinal changes in white matter microstructure do not include additional ROIs.

#### Additional cannabis use characteristics

No significant associations were observed between age of cannabis use onset, chronicity of cannabis use, or past year frequency of use and FA of the cingulum or ATR at age 20.

### Longitudinal association between extended cannabis use and developing anterior cingulate cortex connectivity from age 20 to 22

#### Cingulum

**FIGURE 2:**
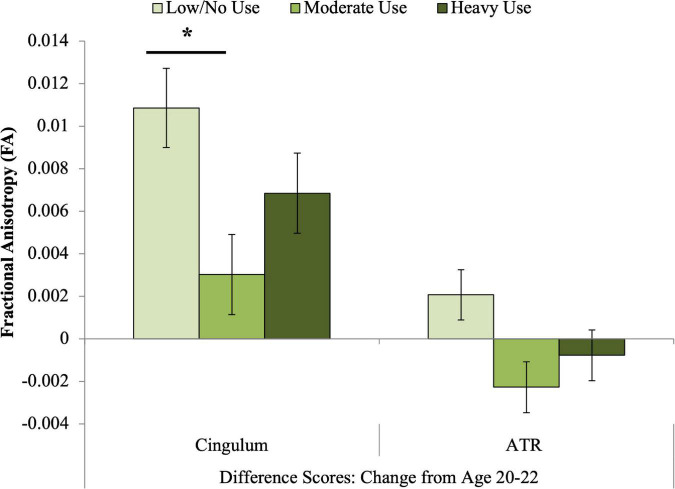
Associations between cannabis exposure and longitudinal FA development from 20 to 22. A significant association was observed between extended cannabis exposure and change in cingulum FA across these 2 years. Bonferroni-corrected *post hoc* tests revealed that the difference between the minimal use and moderate extended cannabis use groups was statistically significant for change in cingulum FA (*p*_corrected_ < 0.05). *indicates significant Bonferroni-corrected *post hoc* pairwise tests.

All extended cannabis use groups displayed increased FA from age 20 to 22, but FA change differed by group across these 2 years (Table 4 and Figure 2). The 2-year increase in FA was significantly larger for the minimal relative to the moderate extended use group (*p*_corrected_ = 0.01). The same pattern of results was found when including only those participants who maintained a consistent level of use across adolescence and the transition to adulthood (*n* = 139, see section “Cannabis use”; Supplementary Table 3).

#### Anterior thalamic radiations

The association between extended cannabis use group and change in ATR FA did not reach our corrected significance threshold (Table 4 and Figure 2).

#### Additional cannabis use characteristics

No significant associations were observed between age of cannabis use onset, chronicity of cannabis use, or past year frequency of use and change in FA of the cingulum or ATR from age 20 to 22.

### Sensitivity analyses

#### Tobacco

Independently, smoking status was not significantly associated with age 20 FA of the cingulum or ATR or change in FA of the cingulum or ATR from age 20 to 22 (see Supplementary Table 4). When controlling for smoking status, we found the same pattern of results: adolescent cannabis use group was a significant predictor of age 20 FA of the cingulum and ATR, and extended cannabis use group was significantly associated with change in FA from ages 20 to 22 in the cingulum, whereas this association only reached trend-level significance in the ATR. The association with smoking status was not significant in any of these models (see Table 5).

**TABLE 5:** Sensitivity analyses for smoking status.

	Cingulum			ATR		
		
	*F*	*df*	*p*	*F*	*df*	*p*
**Results controlling for smoking status**						
**Age 20 FA^•^**						
Adolescent cannabis use group	**3.89**	**2**	**0.021**	**6.2**	**2**	**0.002**
Hemisphere	**12.11**	**1**	**<0.001**	**15.77**	**1**	**<0.001**
Head motion (age 20)	**6.71**	**1**	**0.01**	0.35	1	0.552
Daily smoker (age 20)	3.71	1	0.055	0.34	1	0.558
**Change in FA from 20 to 22^••^**						
Extended cannabis use group	**3.88**	**2**	**0.022**	*2.61*	*2*	*0.075*
Hemisphere	**15.51**	**1**	**<0.001**	2.66	1	0.104
Head motion (age 20)	2.97	1	0.086	1.42	1	0.234
Head motion (age 22)	1.86	1	0.173	1.25	1	0.264
Daily smoker (age 22)	0.94	1	0.333	2.37	1	0.124
**Results excluding daily smokers (remaining n = 112)**						
**Age 20 FA°**						
Adolescent cannabis use group	*3.6*	*2*	*0.029*	*3.75*	*2*	*0.025*
Hemisphere	**7.07**	**1**	**0.008**	**10.76**	**1**	**0.001**
Head motion (age 20)	**6.96**	**1**	**0.009**	0.04	1	0.835
**Change in FA from 20 to 22°°**						
Extended cannabis use group	*2.74*	*2*	*0.067*	*3.67*	*2*	*0.027*
Hemisphere	**10.62**	**1**	**0.001**	1.77	1	0.184
Head motion (age 20)	0.938	1	0.334	2.05	1	0.153
Head motion (age 22)	0.515	1	0.474	1.9	1	0.169

Each quadrant represents one ANCOVA and significant associations (*p* < 0.025) are bolded. Associations that only reach trend-level significance after accounting for smoking status are italicized. ^•^df_error_ = 306, df_total_ = 312; ^••^df_error_ = 309, df_total_ = 316; °df_error_ = 219, df_total_ = 224; °°df_error_ = 218, df_total_ = 224. Change in FA from 20 to 22 represents the difference score for FA from age 20 to 22. FA, fractional anisotropy; ATR, anterior thalamic radiations; *df*, degrees of freedom.

Similarly, when we repeated our primary analyses excluding all daily smokers (remaining *n* = 112), we again saw the same pattern of results, although the association only reached trend-level significance in this reduced sample (see Table 5).

#### Alcohol

Independently, greater alcohol exposure was associated with reduced FA of the cingulum at age 20. Alcohol exposure was not significantly associated with age 20 FA of the ATR or change in FA from ages 20 to 22 for the cingulum or ATR (see Supplementary Table 5). When controlling for cumulative alcohol exposure, we again observed the same pattern of results. Adolescent cannabis use group remained a significant predictor of age 20 FA of the cingulum and ATR, and extended cannabis use group was significantly associated with change in FA from ages 20 to 22 in the cingulum, whereas this association only reached trend-level significance in the ATR. The association between alcohol exposure and cingulum FA at age 20 was significant, with greater exposure associated with reduced FA in this tract. The association between alcohol exposure and FA was not significant in the remaining models (see Table 6).

**TABLE 6:** Sensitivity analyses for alcohol exposure.

	Cingulum			ATR		
		
	*F*	*df*	*p*	*F*	*df*	*p*
**Results controlling for alcohol exposure**						
**Age 20 FA^•^**						
Adolescent cannabis use group	**7.85**	**2**	**<0.001**	**8.38**	**2**	**<0.001**
Hemisphere	**11.69**	**1**	**<0.001**	**14.53**	**1**	**<0.001**
Head motion (age 20)	**7.01**	**1**	**0.009**	0.37	1	0.541
Cumulative alcohol exposure (age 13–19)	**13.51**	**1**	**<0.001**	0.65	1	0.422
**Change in FA from 20 to 22^••^**						
Extended cannabis use group	**4.63**	**2**	**0.01**	*2.53*	*2*	*0.082*
Hemisphere	**15.5**	**1**	**<0.001**	2.64	1	0.105
Head motion (age 20)	3.41	1	0.066	1.41	1	0.235
Head motion (age 22)	2.23	1	0.136	1.27	1	0.26
Cumulative alcohol exposure (age 13–21)	0.66	1	0.417	0.07	1	0.791

Each quadrant represents one ANCOVA and significant associations (*p* < 0.025) are bolded. Associations that only reach trend-level significance after accounting for alcohol exposure are italicized. ^•^df_error_ = 298, df_total_ = 304; ^••^df_error_ = 309, df_total_ = 316. Change in FA from 20 to 22 represents the difference score for FA from age 20 to 22. FA, fractional anisotropy; ATR, anterior thalamic radiations; *df*, degrees of freedom.

## Discussion

The current study aimed to examine the relationship between cannabis exposure and developing ACC connectivity during the transition to adulthood. Contrary to our expectations, moderate, but not heavy, adolescent cannabis users displayed higher FA in the cingulum and ATR relative to minimal users at age 20, even after controlling for tobacco and alcohol exposure. In contrast, our longitudinal results supported our hypothesis that cannabis exposure is associated with reduced white matter maturation – attenuated increase in cingulum FA – from ages 20 to 22. Among minimal extended cannabis users, FA of the cingulum and ATR increased across this 2-year period – consistent with normative development of these pathways – but this increase in white matter integrity of the cingulum was reduced among moderate and heavy extended cannabis users. These results align with existing longitudinal studies (Bava et al., 2013; Jacobus et al., 2013a,b; Becker et al., 2015), and collectively provide evidence that cannabis exposure during adolescence and the transition to adulthood is associated with diminished white matter maturation of the cingulum.

Taken together, the results of our cross-sectional and longitudinal analyses highlight the need to distinguish premorbid neural characteristics associated with risk of use from the neurobiological effects of cannabis exposure. However, a variety of different patterns of aberrant white matter development may contribute to risk for psychopathology (Hulvershorn et al., 2014), and both delayed (Acheson et al., 2014a,b) and accelerated (Squeglia et al., 2014) patterns of white matter development have been identified among individuals at high familial risk for substance use. There are multiple possible paths to substance use and multiple outcomes resulting from use, as both early-developing and late-developing white matter microstructure may each contribute differently to increased propensity for substance use and the development of related problems.

One possible interpretation of the current findings is that accelerated white matter development could be a characteristic of those who are on a steeper developmental trajectory, which may be linked to heightened risk for substance use. Of particular relevance to the current sample, Belsky’s fast-life theory of socialization, based on evolutionary models, proposes that familial psychosocial stress leads individuals to mature more rapidly and reproduce earlier to improve their reproductive fitness within insecure environments (Hochberg and Belsky, 2013). Congruently, emerging animal and human literature suggests that early adversity may accelerate the development of cortical-subcortical connectivity (McPherson et al., 2013; Gee and Cohodes, 2021), and early pubertal maturation has been linked to more advanced white matter development in late adolescence (Chahal et al., 2018). As participants in the current study were recruited based on low SES, this sample is characterized by high rates of neighborhood impoverishment, low income, and maternal depression, among other sources of childhood adversity (Shaw et al., 2016), which could potentially lead to a compensatory acceleration in white matter development. In turn, precocious white matter development may contribute to earlier autonomy, exploration, and socialization (Squeglia et al., 2014). Indeed, prior research has reported higher ATR FA to be linked to heightened risky behavior among adolescents, based on both self-report (Berns et al., 2009) and behavioral measures (Kwon et al., 2014).

**FIGURE 3:**
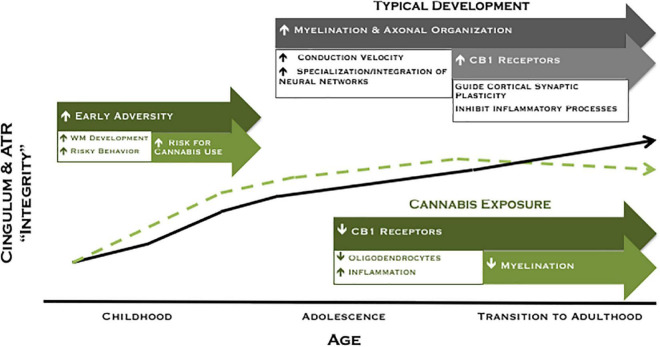
Revised theoretical model of developing ACC connectivity among individuals with and without meaningful cannabis exposure. In typical development (black line), increased myelination, axonal organization, and CB1 receptor expression are postulated to give rise to increased white matter integrity across adolescence and into adulthood. Exposure to adversity early in development may lead to a compensatory acceleration of white matter development, which may increase risky behavior and risk for cannabis use among a subset of individuals (green dashed line). Conversely, cannabis exposure is associated with reduced white matter maturation of the cingulum during the transition to adulthood, an effect that may be mediated by a downregulation of CB1 receptor expression and/or direct effects on oligodendrocyte survival and myelination.

Collectively, our findings of higher FA among moderate cannabis users at age 20 and decreased FA for users across 2 years suggest a pattern of white matter development in which a subset of participants are characterized by higher FA prior to the onset of cannabis use (potentially attributable to early adversity) that may increase their liability to experiment with drugs, followed by reduced white matter maturation with extended cannabis use. We have illustrated this theoretical model in Figure 3. Accordingly, higher FA may represent a marker of risk, whereas cannabis exposure is associated with poorer white matter integrity over time. Although speculative, the current model provides promising avenues for future research to disentangle neural risk factors from cannabis effects on the developing brain.

Associations between cannabis use and white matter microstructure varied substantially between the moderate and heavy cannabis use groups, suggesting an important role of dose/use characteristics. At age 20, moderate adolescent users displayed the highest FA in both pathways, and the moderate extended cannabis use group was characterized by a more substantial reduction in white matter development from ages 20 to 22 relative to the heavy extended use group. To our knowledge, no previous studies have compared white matter development between cannabis users with different levels of use. However, to speculate about what may be driving this pattern, the heavy users could be further along the cannabis exposure trajectory of white matter development illustrated in Figure 3. Indeed, the heavy extended use group initiated cannabis use earlier (mean age 14.7 versus 16.1) and used more frequently than the moderate extended use group. This pattern of results is also interesting in light of previous findings from the same sample that an escalating trajectory of cannabis use across adolescence was associated with altered functional connectivity, relative to both stable low and stable high use trajectories (Lichenstein et al., 2017). Collectively, these studies highlight the importance of considering cannabis use characteristics – including dose, timing, and trajectory – to better characterize the effects of cannabis exposure on the developing brain.

The time course of cannabis effects on the brain remains poorly understood, although data on CB1 receptor changes with cannabis use are informative. Chronic cannabis use has been linked to a downregulation of CB1 receptors (Hirvonen et al., 2012). However, this finding was based on a case-control study including daily cannabis smokers who had been using for a mean of 12 years (Hirvonen et al., 2012). Therefore, it is unclear whether this effect occurs quickly and is then sustained, or if it occurs gradually over the course of many years of exposure. Nonetheless, follow-up data demonstrated that receptor levels normalized after ∼4 weeks of abstinence (Hirvonen et al., 2012), suggesting that the downregulation in receptor expression takes place on a timescale of weeks to months, not gradually over years. Therefore, cannabis effects may plateau with protracted use, which could be reflected in the current pattern of results. Accordingly, cannabis effects on change in white matter microstructure may have been more robust among moderate cannabis users because the heavy users are at a later point on the trajectory when cannabis effects have begun to plateau.

Notably, we did not find significant associations between age of cannabis use onset, chronicity of use, or recent frequency of use and FA of the cingulum and ATR at age 20 or change in FA from ages 20 to 22. These findings suggest that it may be critical to consider the overall quantity of exposure across development to understand the link between cannabis use and microstructure of WM pathways underlying ACC connectivity during the transition to adulthood. The lack of repeated follow-up assessments in prior research may partially explain inconsistent findings in the literature assessing links between cannabis use and white matter integrity, as the majority of this literature has relied on case-control designs (users versus non-users) rather than assessing patterns of use across time (Lichenstein et al., 2022).

It is also important to consider other characteristics that differ among the cannabis use groups that may contribute to the differences in FA of the cingulum and ATR observed. Indeed, indices of SES did differ significantly between groups: the moderate cannabis exposure group was characterized by the highest family income during early childhood, followed by the minimal exposure group and the heavy exposure group. Additionally, the heavy cannabis exposure group was characterized by higher neighborhood risk relative to the minimal exposure group. Prior research has found higher SES to be related to improvements in measures of white matter integrity (Gianaros et al., 2013). Nonetheless, comparing models with and without SES included as a covariate, we found that the inclusion of SES did not significantly improve the model fit for either our cross-sectional or longitudinal analyses. Therefore, there is not direct evidence to suggest that differences in SES are driving the current pattern of results. However, future research will be needed to disentangle independent and interactive effects of cannabis exposure and SES on white matter maturation.

Although the current study has many strengths, including prospective, longitudinal data on adolescent/emerging adult cannabis use and white matter microstructure in a large sample of high-risk young men, there are also several limitations. We investigated a population at high risk for both cannabis use and its adverse consequences (Martin et al., 2015), but our results may not be generalizable to women, individuals of higher SES, or participants from suburban or rural communities. Additionally, the current study relies on retrospective self-reports of cannabis use. Future studies would benefit from estimates from multiple sources, prospective measurement of use, and details on cannabinoid composition (Batalla et al., 2013; Lorenzetti et al., 2016; Mandelbaum and de la Monte, 2017). Finally, although TBSS is relatively robust to the effects of fiber anatomy, metrics derived from the tensor model are highly susceptible to distortion from complex fiber geometry (Concha, 2014). Optimally, as with the ongoing large-scale Adolescent Brain Cognitive Development study (ABCD^1^), we will learn about altered pattern and pace of white matter maturation in cannabis users through investigations that use prospective, longitudinal designs, with detailed measurements of individuals’ social, cultural, and developmental context. Additionally, smaller-scale studies that measure both cannabis use and white matter development more frequently (e.g., dense sampling) can provide improved temporal resolution to elucidate cannabis effects on white matter microstructure over the short-term (e.g., weeks to months versus years).

## Conclusion

Cannabis use is common during adolescence and the transition to adulthood. Although often considered benign, cannabis use has been associated with a wide array of negative outcomes that can have profound impacts on individuals’ long-term trajectory of achievement, health, and wellbeing. However, the neurobiological mechanisms that underlie the deleterious effects of cannabis exposure, especially at vulnerable developmental periods and in high-risk populations, remain poorly understood. The current study used longitudinal DTI data to demonstrate that cannabis use is associated with lesser white matter maturation of the cingulum from ages 20 to 22. These results have important implications for understanding cannabis effects on brain structure and function and informing public perceptions about the risks of cannabis use. Elucidating the neural basis of cannabis effects can facilitate the development of targeted prevention and intervention strategies to foster positive development among individuals at highest risk for cannabis use and poor psychosocial adjustment.

## Data availability statement

Data were acquired as part of the Pitt Mother and Child Project. Access is restricted to protect patient confidentiality and participant privacy. Requests to access these datasets should be directed to DS, danielshaw@pitt.edu.

## Ethics statement

The studies involving human participants were reviewed and approved by the Institutional Review Board at the University of Pittsburgh. Written informed consent to participate in this study was provided by the participants’ legal guardian/next of kin.

## Author contributions

SL and EF contributed to conception and design of the current study. DS was MPI of the Pitt Mother and Child Project (PMCP), the parent study from which the current data were drawn and oversaw all aspects of PMCP study design and data collection. SL performed the statistical analysis and wrote the first draft of the manuscript. All authors contributed to manuscript revision, read, and approved the submitted version.

## Footnotes

1
https://abcdstudy.org/
