# Supplementary material for: Cannabis, connectivity, and coming of age: Associations between cannabis use and anterior cingulate cortex connectivity during the transition to adulthood
Source: Front Hum Neurosci. 2022 Nov 11;16:951204. doi: 10.3389/fnhum.2022.951204 (PMC9692120; doi:10.3389/fnhum.2022.951204)
Supplement: Supplementary file 1 [file Data_Sheet_1.DOCX]

Supplementary Material

# Supplementary Table 1. Model Selection

| **Difference Between Partial Model AIC and AIC of Model including Control Variable** | | | | | |  |
| --- | --- | --- | --- | --- | --- | --- |
|  | SES | IQ | Internalizing | Externalizing | Tobacco | Alcohol |
| **Age 20 FA** |  |  |  |  |  |  |
| Right Cingulum | -14.04 | -194.84 | -112.96 | -114.66 | -13.23 | -30.27 |
| Left Cingulum | -17.64 | -203.79 | -122.96 | -123.48 | -10.83 | -37.36 |
| Right ATR | -23.42 | -231.56 | -142.25 | -144.60 | -19.54 | -52.29 |
| Left ATR | -23.71 | -231.80 | -142.95 | -144.56 | -19.52 | -48.74 |
| **Change in FA from Age 20 to 22** | |  |  |  |  |  |
| Right Cingulum | -27.33 | -223.22 | -141.17 | -146.27 | -17.00 | -33.86 |
| Left Cingulum | -29.14 | -242.91 | -149.92 | -151.55 | -19.60 | -44.24 |
| Right ATR | -30.24 | -263.88 | -156.36 | -158.87 | -20.48 | -43.31 |
| Left ATR | -30.45 | -247.19 | -149.51 | -151.76 | -18.62 | -43.17 |

*Note.* Akaike Information Criterion (AIC) values were calculated for regression models predicting FA of the right and left cingulum and ATR based on adolescent cannabis exposure, controlling for head motion during DTI scanning. These were compared to AIC values for regression models including each of the potential covariates. Smaller AIC values reflect better regression model fit. Therefore, the AIC values from the models including our control variables were subtracted from the AIC value of the original model. Difference values greater than 2 reflect a substantial improvement in model fit. No control variables were found to significantly improve model fit; therefore, only hemisphere and head motion were included as covariates in primary models.

# Supplementary Table 2. Associations between Cannabis Use and MD, RD, and AD of the Cingulum and ATR

|  | Cingulum | | |  | ATR | | |
| --- | --- | --- | --- | --- | --- | --- | --- |
|  | *F* | *df* | *p* |  | *F* | *df* | *p* |
|  | Age 20 MD | | | | | | |
| Adolescent Cannabis Use Group | **7.39** | **2** | **<0.001** |  | 3.48 | 2 | 0.032 |
| Hemisphere | **12.72** | **1** | **<0.001** |  | **8.19** | **1** | **0.004** |
| Head Motion | 0.896 | 1 | 0.345 |  | 0.093 | 1 | 0.76 |
|  | Age 20 RD | | | | | | |
| Adolescent Cannabis Use Group | **3.8** | **2** | **0.023** |  | 2.53 | 2 | 0.081 |
| Hemisphere | 0.05 | 1 | 0.823 |  | **15.01** | **1** | **<0.001** |
| Head Motion | 0.041 | 1 | 0.841 |  | 0.152 | 1 | 0.697 |
|  | Age 20 AD | | | | | | |
| Adolescent Cannabis Use Group | 2.8 | 2 | 0.063 |  | **4.09** | **2** | **0.018** |
| Hemisphere | **28.25** | **1** | **<0.001** |  | 0.369 | 1 | 0.544 |
| Head Motion | 1.48 | 1 | 0.225 |  | 1.44 | 1 | 0.231 |
|  | Change in MD from 20 to 22 | | | | | | |
| Extended Cannabis Use Group | 0.23 | 2 | 0.798 |  | 1.74 | 2 | 0.178 |
| Hemisphere | 0.49 | 1 | 0.486 |  | 0.276 | 1 | 0.6 |
| Head Motion (age 20) | 0.2 | 1 | 0.657 |  | 3.25 | 1 | 0.072 |
| Head Motion (age 22) | 0.09 | 1 | 0.763 |  | 3.57 | 1 | 0.06 |
|  | Change in RD from 20 to 22 | | | | | | |
| Extended Cannabis Use Group | 1.32 | 2 | 0.268 |  | 2.77 | 2 | 0.064 |
| Hemisphere | 0.43 | 1 | 0.512 |  | 0.00 | 1 | 1.000 |
| Head Motion (age 20) | 0.63 | 1 | 0.429 |  | 0.00 | 1 | 0.955 |
| Head Motion (age 22) | 0.75 | 1 | 0.386 |  | 0.41 | 1 | 0.523 |
|  | Change in AD from 20 to 22 | | | | | | |
| Extended Cannabis Use Group | 0.74 | 2 | 0.477 |  | 0.07 | 2 | 0.931 |
| Hemisphere | 0.02 | 1 | 0.899 |  | 0.05 | 1 | 0.823 |
| Head Motion (age 20) | 0.18 | 1 | 0.671 |  | **8.33** | **1** | **0.004** |
| Head Motion (age 22) | 2.87 | 1 | 0.091 |  | **12.34** | **1** | **<0.001** |

*Note*. Each quadrant represents one ANCOVA and significant effects (*p*<.025) are bolded. Change in MD, RD and AD from 20 to 22 represents the difference score rom age 20 to 22. ATR=anterior thalamic radiations, *df*=degrees of freedom MD=mean diffusivity, RD=radial diffusivity, AD=axial diffusivity.

# Supplementary Table 3. Results including Stable Users Only (n=139)

|  | Cingulum | | |  | ATR | | |
| --- | --- | --- | --- | --- | --- | --- | --- |
|  | *F* | *df* | *p* |  | *F* | *df* | *p* |
|  | Change in FA from 20 to 22 | | | | | | |
| Extended Cannabis Use Group | **3.81** | **2** | **0.024** |  | *2.76* | *2* | *0.065* |
| Hemisphere | **11.23** | **1** | **<0.001** |  | 1.38 | 1 | 0.242 |
| Head Motion (age 20) | 2.6 | 1 | 0.108 |  | 1.69 | 1 | 0.195 |
| Head Motion (age 22) | 1.98 | 1 | 0.16 |  | 1.87 | 1 | 0.173 |

*Note*. Each quadrant represents one ANCOVA and significant effects (*p*<.025) are bolded. Effects that only reach trend-level significance after excluding participants whose level of use changed between adolescence and the transition to adulthood are italicized. Change in FA from 20 to 22 represents the difference score for FA from age 20 to 22. FA=fractional anisotropy, ATR=anterior thalamic radiations, *df*=degrees of freedom.

# Supplementary Table 4. Independent Effects of Smoking Status

|  | Cingulum | | |  | ATR | | |
| --- | --- | --- | --- | --- | --- | --- | --- |
|  | *F* | *df* | *p* |  | *F* | *df* | *p* |
|  | Age 20 FA | | | | | | |
| Daily Smoker (age 20) | 2.97 | 1 | 0.086 |  | 0.38 | 1 | 0.54 |
| Hemisphere | **11.89** | **1** | **<0.001** |  | **15.25** | **1** | **<0.001** |
| Head Motion | 7.3 | 1 | 0.007 |  | 0.09 | 1 | 0.767 |
|  | Change in FA from 20 to 22 | | | | | | |
| Daily Smoker (age 22) | 1.99 | 1 | 0.16 |  | 4.1 | 1 | 0.044 |
| Hemisphere | **15.23** | **1** | **<0.001** |  | 2.63 | 1 | 0.106 |
| Head Motion (age 20) | 2.4 | 1 | 0.122 |  | 1.11 | 1 | 0.293 |
| Head Motion (age 22) | 1.29 | 1 | 0.258 |  | 0.88 | 1 | 0.35 |

*Note*. Each quadrant represents one ANCOVA and significant effects (*p*<.025) are bolded. Change in FA from 20 to 22 represents the difference score for FA from age 20 to 22. FA=fractional anisotropy, ATR=anterior thalamic radiations, *df*=degrees of freedom.

# Supplementary Table 5. Independent Effects of Alcohol Exposure

|  | Cingulum | |  | ATR | |
| --- | --- | --- | --- | --- | --- |
|  | *β* | *p* |  | *β* | *p* |
|  | Age 20 FA | | | | |
| Cumulative Alcohol Exposure (age 13-19) | **-0.14** | **0.01** |  | -0.02 | 0.666 |
| Hemisphere | **0.185** | **<.001** |  | **-3.72** | **<.001** |
| Head Motion | **-0.143** | **0.01** |  | 0.02 | 0.785 |
|  | Change in FA from 20 to 22 | | | | |
| Cumulative Alcohol Exposure (age 13-21) | -0.03 | 0.63 |  | -0.08 | 0.166 |
| Hemisphere | **-0.21** | **<.001** |  | -0.09 | 0.107 |
| Head Motion (age 20) | 0.26 | 0.13 |  | 0.17 | 0.343 |
| Head Motion (age 22) | -1.15 | 0.253 |  | -0.16 | 0.379 |

*Note*. Each quadrant represents one regression model and significant effects (*p*<.025) are bolded. Change in FA from 20 to 22 represents the difference score for FA from age 20 to 22. FA=fractional anisotropy, ATR=anterior thalamic radiations.
